# Co-producing Psychiatric Education with Service User Educators: a Collective Autobiographical Case Study of the Meaning, Ethics, and Importance of Payment

**DOI:** 10.1007/s40596-019-01160-5

**Published:** 2019-12-23

**Authors:** Sophie Soklaridis, Alise de Bie, Rachel Beth Cooper, Kim McCullough, Brenda McGovern, Michaela Beder, Gail Bellissimo, Tucker Gordon, Suze Berkhout, Mark Fefergrad, Andrew Johnson, Csilla Kalocsai, Sean Kidd, Nancy McNaughton, Charlotte Ringsted, David Wiljer, Sacha Agrawal

**Affiliations:** 1 The Centre for Addiction and Mental Health, Toronto, ON Canada; 2 McMaster University, Hamilton, ON Canada; 3 University of Toronto, Toronto, ON Canada; 4 Wilfred Laurier University, Waterloo, ON Canada; 5 1353 Danforth Ave, suite #2, Toronto, M4J 1N1 Ontario Canada; 6 Unity Health Toronto, Toronto, ON Canada; 7 2548 Strathmore Crescent, Mississauga, L5M 5L1 Ontario Canada; 8 Sunnybrook Health Sciences Centre, Toronto, ON Canada; 9 University Health Network, Toronto, ON Canada; 10 Aarhus University, Aarhus, Denmark

**Keywords:** Co-production, Service user educator, Mental health education, Payment, Qualitative research

## Abstract

**Objective:**

Co-production involves service providers and service users collaborating to design and deliver services together and is gaining attention as a means to improve provision of care. Aiming to extend this model to an educational context, the authors assembled a diverse group to develop co-produced education for psychiatry residents and medical students at the University of Toronto over several years. The authors describe the dynamics involved in co-producing psychiatric education as experienced in their work.

**Methods:**

A collaborative autobiographical case study approach provides a snapshot of the collective experiences of working to write a manuscript about paying service users for their contributions to co-produced education. Data were collected from two in-person meetings, personal communications, emails, and online comments to capture the fullest possible range of perspectives from the group about payment.

**Results:**

The juxtaposition of the vision for an inclusive process against the budgetary constraints that the authors faced led them to reflect deeply on the many meanings of paying service user educators for their contributions to academic initiatives. These reflections revealed that payment had implications at personal, organizational, and social levels.

**Conclusion:**

Paying mental health service user educators for their contributions is an ethical imperative for the authors. However, unless payment is accompanied by other forms of demonstrating respect, it aligns with organizational structures and practices, and it is connected to a larger goal of achieving social justice, the role of service users as legitimate knowers and educators and ultimately their impact on learners will be limited.

The co-production of psychiatric services involves service providers, service users, family members, and carers working together to create services that better meet the needs of all stakeholder parties [1, 2]. The approach is value driven and built on the principle that those who use a service are best placed to help design it. Co-production moves beyond a consultation model or the involvement of service users for education in relatively circumscribed, pre-determined roles by engaging service users from the very beginning of an educational initiative so that it can be designed and delivered together with service providers. Key principles of co-production include breaking down the barriers between service providers and users, building on peoples’ existing capabilities, reciprocity, acknowledging peer and personal support networks, and facilitating a shift in services toward change agency. Throughout the process, the perspectives of service users are respected and incorporated in meaningful and mutually agreed upon ways. Working with people who are most directly affected by health services to design and deliver services together fits closely with contemporary ideas about patient- and person-centered care [3] and patient engagement [4] and is increasingly seen as a desirable feature of recovery-oriented mental health care and research [5]. However, the analogous practice of co-producing psychiatric *education*, in which psychiatric educators and service user educators design and deliver psychiatric training together, is still relatively unexplored. In contrast to other health professions within mental health care, such as nursing and social work, and some other branches of medicine, psychiatry has been slow to adopt this educational practice [6–11].

One reason for this discrepancy may be that co-producing psychiatric education is different in important ways from co-producing professional education in other health disciplines. First, psychiatrists have a distinctive role of diagnosing mental illness, which can have both helpful and harmful consequences for service users, given the high levels of prejudice and discrimination that people with mental health challenges frequently experience [12]. Second, psychiatrists have the legal authority to admit people to psychiatric facilities and to give treatment without their consent, a power that can work against the collaboration required for co-production. Third, a particularly stark difference in social position often exists between psychiatrists and service users due to the marginalization that many people with mental health challenges experience because of reduced access to education, income, employment, and dignified housing [13]. Thus, the social landscape in which psychiatry operates poses multiple challenges to the principles of co-production, which include breaking down distinctions between service users and service providers, building on service users’ strengths and capabilities, and operating in a way that creates opportunities for reciprocal learning and mutual benefits [1].

We can learn from the experiences and pedagogies [14] of psychiatric survivors [15], people with disabilities [16], and Indigenous communities [17] as they have advocated for the recognition that their members hold legitimate knowledge and expertise that are valuable in their own right. While there are many differences between and within these communities, they share a common goal of liberation from social injustices, including *epistemic injustice*, which refers to the harms done when people are unfairly reduced as non-knowers, often because of prejudice [18]. Epistemic injustice occurs when an individual’s experience is discounted and not given the appropriate amount of credibility by persons in position of power who are considered to be the legitimate knowers. These perspectives emphasize the importance for members of powerful institutions (such as physicians and researchers) to acknowledge historical injustices, practice cultural humility, and engage in continuous reflection and reflexivity when seeking to work and collaborate with historically marginalized groups.

Notwithstanding these challenges, interest in the idea of bringing the unique knowledge and perspectives of service users directly into training is growing in psychiatry [19]. For example, the Royal College of Psychiatrists mandated in 2005 that UK residents receive training from service users [20]. More recently, the landmark World Psychiatric Association–Lancet Psychiatric Commission on the Future of Psychiatry [21] found that “Incorporation of patients (and carers) as educators within medical training is particularly important to teach the principles of recovery-oriented care and combat negative stereotypes of patients with mental illness and substance use disorders.” Published reports of co-produced education in psychiatry remain scarce, however, and little is known about how co-production in psychiatric education actually plays out or how it can best be implemented [22–24].

With these ideas in mind, a group of educators situated in and near the University of Toronto aimed to build capacity to co-produce education for its psychiatry residents and medical students. A provincial health innovation grant allowed us to create a working group of local educators who came from diverse social backgrounds and included people who have been marginalized due to race, gender, class, sexual orientation, and Indigenous identity, as well as people with lived experience of mental health and substance use challenges, community advocates and activists, education researchers, trainees, and psychiatrists. The group met monthly and was co-chaired by a psychiatrist (SA), who was also the principal investigator of the grant, and a service user educator (KM). The grant had three objectives: (1) to develop local opportunities for co-produced psychiatric education; (2) to build capacity by developing a series of workshops and a community of practice for service user educators; and (3) to examine peoples’ experiences of doing this work together and to disseminate the lessons learned as a set of “best practices.” We conceived this paper with this last objective in mind.

As we embarked on the task of disseminating some of the lessons learned working together through several collaborative projects over the years, we soon discovered that the very act of writing this manuscript brought with it the same complex dynamics that co-producing psychiatric education frequently entails. Questions surfaced about the way power was playing out in our writing project, the presence of competing and hidden agendas, and the risks of tokenism and exploitation, all themes that we had come to understand as critical issues in co-produced education. Thus, rather than summarize and reflect on our educational projects, we have taken the writing of this manuscript itself as the object of study. More specifically, because this writing project was only modestly funded, and our financial constraints posed significant challenges to our collaboration, we use here the notion of paying service users for their contributions to co-produced academic work as an entry point into these complex dynamics. We use a collaborative autobiographical case study to examine the issue of payment at three overlapping levels, personal, organizational, and social, and we offer reflective questions at the end of each theme for the reader to consider. We also explore how payment reflects broader considerations related to value, power, and justice.

## Methods

We used a modified reflective topical autobiographical approach to this work, which focuses on a snapshot of the person’s story that is of some topical interest [25–27]. In this article, because none of the stories were specifically autobiographical, we give readers a snapshot of our collective experiences of working together to write a manuscript about payment in co-production (the case). We thus invite others who are engaged in related activities to build a “shareable understanding” of co-production ([25], p. 28). Our approach can best be described as a collaborative autobiographical case study approach.

We sent an email to every member of the working group and to others who have worked closely on one or more of our co-produced initiatives, including a novel service user advisory course for residents [12, 16], an initiative to include service users in recruitment and selection for our psychiatry residency program, and new service user-led activities for medical students, to invite them to join the manuscript writing process if they were interested, willing, and able. Those who agreed to be part of the manuscript writing process are the authors of this paper. Consistent with the principle of co-production of breaking down false and misleading barriers between service users and providers, we deliberately did not identify whether members of our writing group have experienced mental health challenges or if any of us have used mental health services.

### Data Collection and Analysis

We held two in-person manuscript writing meetings several months apart. Each meeting had a recorder (SS) who typed out the discussion in real time. Every effort was made to capture the conversations verbatim. Throughout the writing process, we subsequently communicated in person and through email and used Google Docs to share feedback on various outlines and drafts as we explored, described, and problematized co-produced education.

When we initially set out to draft our best practices article, many of us felt disengaged and uninspired. Through email and personal communication, we explored possible sources for these feelings. For some, the problem resided in the language of “best practice,” which seemed to imply a static and universal set of rules to guide co-production, whereas we felt that co-production demanded a flexible, negotiated, and local approach. For others, the arrogance suggested by authoring a “best” statement felt uncomfortable. Logistical constraints such as page limits and number of citations allowed in the “perspectives” genre were also discouraging and would have forced us to omit important sources that we drew upon in our work.

Another source of dissatisfaction was the manuscript writing process itself, which some authors perceived to emphasize simplicity and expediency over complexity and nuance. The politics of traditional manuscript writing tend to serve the purpose of publishing academic work as quickly as possible and do not allow authors to take time to reflect deeply on their identities and their experiences when multiple, sometimes conflicting, viewpoints are held. It seemed problematic that we were inadvertently shortcutting our own prior slow, careful process at the risk of glossing over important perspectives. One author (RBC) noted in an email, “I wonder if there’s an implicit wish to hurry this process along to publish and “get ‘er done” without actually unpacking what it means to do just that.”

Much of the “unpacking” was done in between the group’s scheduled formal communication. For example, RBC and ABD on a long walk discussed various topics related to co-production that precipitated the email above. RBC and SS were at another event, and they too had discussions prompted by the email about the perceived time pressure to publish this work. In addition to coordinating author schedules and managing technology to facilitate remote participation for those who were unable to attend meetings in person, we also had ongoing discussions about how long the writing process would take and whether the end product of a manuscript was even a desirable goal, given ambivalence about the process. One author (ABD) suggested an alternative to the “perspective” genre, a manuscript that used the format of a traditional empirical report while “bending the rules,” thus communicating implicitly the importance of finding courage to do things differently: “We could follow the norm, but subvert it all the same by the kinds of things we count as method/result, and the more personal way we read ourselves and our voice into the writing and the work.”

We therefore agreed to change direction toward something more reflexive, an article that took the process by which it was written itself as the object of study. We aim to give voice to our individual experiences and perspectives by using direct quotations. We intentionally identify ourselves in the quotations to challenge how research can be seen to appropriate the knowledge of “subjects” by anonymizing them.

Email communications were unusually rich, perhaps because this format provided a space for additional reflection. Emails to the whole group were sometimes perceived as “risky,” so subgroups, especially of service users and people who had closer relationships with one another, formed. This created a sense of safety for expressing things that were uncomfortable or that were at odds with the views of others. As one author (BM) stated in an email to a subset of the group: “I’m responding to a small group of you as I’m not comfortable sharing with the entire group and I need to make this okay. I’m not comfortable with Google Docs but more importantly I was uncomfortable sharing with the large group by email.”

To capture the fullest range of perspectives on the issue of paying service users, direct quotations for this article were drawn from our two in-person meetings, phone conversations, emails, informal comments at other meetings, and online comments that were made on earlier drafts of the article. The quotations were checked for accuracy, approved by their sources, and lightly edited for clarity.

### Ethics

Our efforts to avoid causing harm to one another, to work together in ways that are mutually beneficial, to respect one another’s autonomy to make informed decisions about participation, and to strive together for greater justice reflect the central principles of biomedical ethics [28] and were important considerations in our day-to-day work. Indeed, the ethical dimensions of this work go well beyond the procedural ethics [29] of obtaining institutional review board approval or documenting informed consent. In fact, our reflections led us to consider the risks of writing for an academic audience in the first place, whose members have historically excluded service users from knowledge and knowledge production. Author RBC captured this concern: “We’ve been asked … to articulate a process that in many ways counters how things have been done [in the past] but also reflects a more inclusive way of knowing and doing that highlights the tensions of inclusion/exclusion, equity and commitment to values and then writing it in a venue that I perceive as value-muting.”

Put another way, when people’s lived experiences are understood and represented mainly as pathology in medical journals, it can feel like a betrayal of one’s values and community to write for these same journals. Ultimately, however, we decided to submit our manuscript to *Academic Psychiatry*, recognizing that we had a responsibility to stimulate discussion about how service provider and service user educators can engage more collaboratively and constructively to improve mental health professional training.

## Results

Our group held the unanimous belief that service user educators must be paid for their work. Limitations in how this ideal is operationalized were and are inevitable, however. While we have strongly advocated in our work for service users to be paid for their contributions to the planning and delivery of education, we only had enough funds at the end of our grant to provide honoraria for two in-person meetings to support the writing of this manuscript. This limitation meant that most of the work involved in preparing this manuscript was unpaid for those of us who do not have jobs that include or reward writing academic papers. The juxtaposition of that reality and our vision for an inclusive process led us to reflect deeply on the meaning of paying service user educators for their contributions to academic initiatives. These reflections revealed that payment had meaning and implications at many overlapping levels: the personal, the organizational, and the social.

### The Personal Landscape

In our group, the importance of paying service users for their contributions to education was often felt at a deeply personal level and connected to a larger socio-political context. For instance, for some, payment enabled participation in its most basic sense: “There are people sitting at that table who are hungry because they can’t afford enough food” (KM).

Alternatively, payment was understood as a way of acknowledging and valuing service users’ expertise, which can otherwise feel underappreciated: “Living with a condition 24/7/365 often provides a deep understanding of an illness.… Sometimes mechanisms used for coping, for example, can be turned into skills used for insight, relationship building, inquiry, compassion and commitment to change” (GB).

Payment also serves to recognize the emotional labor involved in contributing:

This work is HARD and requires an inordinate amount of emotional labour. Being a person with lived experience that is being invited to tables to help inform institutional, systemic and academic change by drawing upon my lived experience requires another concurrent career in self-care, personal growth and commitment to wellness to ensure that I can contribute in a way that is meaningful. Payment, for me, recognizes not only the contribution I make at the table, but is a small (and totally insufficient) token of recognition of the amount of work I must do in private to be able to show up in public. (RBC)

The corollary is that the absence of payment can trigger intense negative feelings. While transparency about our financial limitations was important, it did not resolve the issue: “I felt a sense of deep hostility about the prospect of spending an hour of unpaid time crafting a respectfully and articulately worded [response] to your inquiry” (RBC).

At the same time, it was important for us to recognize that payment represents just one among other, more subtle benefits of collaborating: “In this group, service-user knowledge is cared about and there is a space where it can be developed. Opportunity to better understand my knowledge as knowledge is front and centre” (ABD). However, payment is insufficient by itself to build equitable collaborations, and, depending on how it is handled, can even increase inequity:

Money is not the only way of showing respect – and if we believe it is, then we miss the importance of considering what respect could and should look like throughout the development of a project. With money, it can feel like because you are being paid, it can relieve the payer from their ethics and responsibility. Once they get what they want and pay me, then my voice is done. That feels like being used. It also feels patronizing not to be asked and to have that decision made for me. I can choose when I’m willing to be used or not and I would rather be asked to participate as a collaborator than not to be asked because there’s no money to pay me. (ABD)

Offering payment can also complicate things in another way:

That we were all sitting around the same table, ostensibly working together as equal partners, while earning substantially different amounts of money doing so, was a problem that was called out at our very first working group meeting. I felt uncomfortable with this arrangement, not just because it pointed to a serious inconsistency in our practice but also because it made me painfully aware of my privilege as a physician. It felt like my income was being questioned – and of course, on some level, it was. (SA)

Fostering more inclusive approaches to co-producing mental health education with service user educators will require individuals who work in academic psychiatry to think about how to fund service user involvement in a way that promotes equity. Helpful strategies for working through these tensions require us to acknowledge the risks and strengthen our capacity for ethical collaborations by openly discussing the power imbalances that are inherent in this work. The following reflective questions can guide these challenging conversations:

- How is payment experienced by participants?
- How does payment influence service user participation?
- How can we ensure that payment does not become a “pay-off” that perpetuates feelings of tokenism or exploitation?

### The Organizational Landscape

The issue of payment also had implications at the level of our organizational landscape, which features a large medical school and psychiatry residency program and its associated academic health science centers. For example, the logistics of offering payment to individuals who had no formal university or hospital affiliation in a way that promoted dignity and did not lead to unintentional threats to housing or social assistance benefits [30] was a persistent challenge:

They initially wanted us to give out gift cards – as though we should be telling people how to spend the little bits of money that we can offer them. Over time, we have advocated for improved accounting policies and procedures that meet the needs of our service users for timely cash payments, while respecting the compliance rules that our institutions have to abide by. Still, the whole scheme has felt precarious. More than once we have been told that we have to stop paying people immediately. (SA)

Just as there were no organizational policies that could facilitate payment at the start of our work, there were also no budget lines to draw from. We find ourselves trying to build co-production collaborations while simultaneously investigating their impact and building a case for their place in psychiatric education, which means that funding for service user contributions has thus far largely come from research grants. Relying on research grants means not having funds to pay service users before funds are awarded or after the project has formally ended. These limitations have a clear negative impact on our ability to collaborate, as SS described:

I’ve got a little less than two weeks to write this grant. I want to engage others but I feel like I have nothing to offer. There are so many constraints. The unrealistic timelines, the jargon used in grant-writing to make us look smart, the way we contort our project ideas to make it palpable to the funders … at least I am getting paid to do this. I have no money to pay family members or clients to participate in this grant-writing process. This weighs heavily on me.… I also know that despite the effort of putting together a grant, the end result is usually rejection. How do you build relationships on that? (SS)

Obtaining sustained operational funding to support service user contributions in our collaborations has been a goal from the outset. Among other advantages, strong organizational support creates the possibility of creating permanent employment positions for service user educators that carry labor rights and benefits such as paid vacation and sick days, thus reducing the precariousness of this work. However, our reflections revealed that obtaining organizational support is not without its own challenges:

Unlike grants, where you get money and then do whatever you set out to do, institutional buy-in means working with an institutional agenda. If we get institutional support to provide us with more secure funding to grow these programs, there will be strings. If we are deeply embedded in and indebted to the institution, how do we preserve our independent and critical stance? (SA)

Thinking practically, it is important to be critical of patient engagement strategies such as co-production, especially those initiated by mainstream organizations such as hospital and funding agencies. Critical questions about when, why now, and who decided to make space for the inclusion of the patient’s voice in health professions education require open and authentic discussions among health professional and service users educators. Consider starting a conversation about co-production with these questions:

- How can we provide payment and provide it in a way that strengthens (rather than weakens) the position of service users in the collaboration?
- What does institutional support for co-producing educational initiatives with services users mean, and what will the organization expect in return?

### The Social Landscape

The issue of paying service users for their educational contributions was also examined through the lens of the larger historical and social context in which our collaboration resides. Author ABD reminded us how in the nineteenth century psychiatric patients at the Ontario Provincial Lunatic Asylum were forced to provide unpaid physical labor for constructing several buildings and the grounds’ 16-ft perimeter wall and for work such as laundry, sewing, and cooking [31]. At the time, leading psychiatrists advanced the theory that chores and physical labor were virtuous and offered inmates a sense of purpose and other unnamed health benefits [32]. In parallel, some contemporary education scholars highlight the value to service users of providing unpaid labor for co-production initiatives in the health professions, while others (including members of our group) recognize labor as labor and consider payment as a necessary step toward establishing fair, respectful and equitable collaborations [33–36]. As author KM explained, “We are also excluded from society when the only way of understanding us is as people in need of rehabilitation/return to normalcy/unable to work vs. human beings that have a right to employment, housing, income, responsibilities, etc.”

Asking for and accepting unpaid labor can therefore be understood as perpetuating the marginalization of people with disabilities by reducing the value of their labor and, ultimately, their knowledge and skills: “There are many transferable skills and surprising insights gleaned from lived experience. [Service users] should be remunerated for the insights and contributions they provide” (GB). This expertise is often multi-faceted for service users who belong to multiple historically marginalized communities, such as those who have been affected by the legacies of economic exploitation [37].

In contrast, participation, even paid participation, can have important negative implications on one’s status within one’s community:

The researchers and those who might be researchers are benefiting the most because they are the ones that are increasing their social capital with their peers and other academics by having this paper published. For me, it does nothing in terms of helping my career because I am not an academic and I am not planning on pursuing an academic career. In fact, by participating in this process, I am decreasing my own social capital among my peers because in a way, I am seen by many in my community as being co-opted by the organization I work in and being appropriated by the researchers I work with. (TG)

Paying service user educators for their work is also a political move that can change common representations of volunteerism. Our group discussed some of the pitfalls associated with participating in other volunteer “opportunities.” For example, breast cancer awareness and fundraising have been criticized as “pinkwashing,” a form of social injustice against women [38] wherein shopping and buying pink ribbon products became a distraction that inadvertently discouraged the public from asking controversial questions [39]. These connections with volunteerism were viewed by our group as a caution to keep our work from losing its critical focus, as SB highlighted: “There has been a mass appropriation of recovery that has been used to forward a neo-liberal agenda. So there is a cautionary tale – will we be someone’s public relations campaign?”

Because co-production aims to unsettle the status quo, engaging authentically in this work means feeling uncomfortable in it. Mental health service users’ lived experiences have been historically deemed as “just stories” that are inferior and unreliable compared to other forms of knowledge [40]. To ethically engage in the work of co-production, we need to constantly ask questions:

- How can payment serve to symbolically break with an historic pattern of excluding service users from positions of legitimate and valuable knowledge?
- How can we leverage this educational initiative to facilitate the larger goal of social justice?
- How can we maintain our critical perspective as co-production is brought into the mainstream?

## Discussion

This collaborative autobiographical case study illustrates some of the complexities of co-produced psychiatric education. Drawing inspiration from colleagues who also see health professions education as a fundamentally socio-political act [41, 42], we have insisted on paying service user educators in our projects. However, paradoxically, the writing of this manuscript was only modestly funded, which tested the very basis of our collaboration and forced us to reflect deeply together on the meaning of payment in co-produced education.

Our reflections reveal that the issue of payment reverberated at many overlapping levels. Payment was often experienced in a deeply personal way both in its presence and in its absence and in the way it brought the challenge of truly valuing each other equally into focus. Payment also highlighted the limitations of organizational structures that often create barriers for building collaborations such as ours, which in turn created opportunities for education and advocacy with our host institutions. Further, payment was seen as part of the larger unfolding social context of our work together, where it seemed to signify a break from the historical exploitation of psychiatric patients on the one hand and contemporary co-optations of service users on the other.

Yet our reflections also revealed that payment is a necessary but insufficient means of achieving the ultimate goal of changing the way learners in psychiatry come to understand and work with service users. For example, if payment is seen as an imperative, the absence of funds can be taken as a reason for not inviting service users to participate at all. Similarly, “paying off” service users can be used as a way of limiting obligations to them and their influence. Payment can also erode the uniquely critical stance that service users bring to education. In sum, if our work co-producing psychiatric education does not broadly challenge the way in which service users are diminished as knowers and marginalized as people—if it does not seek to address epistemic and other social injustices—then our attempts to improve psychiatric education will fail, whether those efforts include payment or not [43].

The struggle for payment has been a long-standing concern for service users [31, 44]. Guidebooks and sample payment policies have been developed in response [45, 46], but these conversations are largely missing in the health professions education literature [10, 47, 48]. In contrast to other educators [49], paying service user educators for their contributions has never felt optional for the authors of this paper. We see paying service user educators for their educational contributions as an ethical imperative that offers a tangible way to promote the inclusion of individuals who may otherwise be unable to participate, to acknowledge the importance and legitimacy of service user educators’ knowledge and skill [34–36, 45, 48, 50], and to begin to address the differences in power and privilege that beset our collaborations.

In addition to reflecting on how to address inequities with co-production, we also discussed how to address inequities in the assigning of credit for this work, which is, after all, a nonfinancial form of payment. How should we measure the importance of contributions when some of us, by virtue of our privileged access to education and employment, find ourselves in a position to spend time on academic work, while others do not? How do we account for the fact that some members needed to step back for periods of time from our work together, sometimes as a result of the emotional impact of doing this work? International guidelines for authorship do not reflect these complexities of co-produced work. In the end, we decided that SS would be first author, given her role in facilitating the writing of the manuscript, and SA would be last author, given his role revising the manuscript and his leadership role in the overall project. We listed all other authors who contributed in alphabetical order.

Our co-production network is still early in its evolution, and much remains to be built, explored, and understood. Our ability to offer payment is imperfect and leaves many gaps. We are often forced to choose between attempting to forge meaningful, equitable partnerships while colluding with and reinforcing oppressive relationships among us by not paying service users for their labor or not collaborating at all. And, as we have found, transparency about the limitations of payment, while important, is not enough. Good intentions and goodwill do not resolve the conflicts and power relations that exist in these collaborations. Nevertheless, we plan in future work to build more opportunities for co-produced education, to develop institutional commitment to fund these initiatives, and to continue to explore the experience and impact of co-produced psychiatric education on learners, educators, and systems.
