# Correction to: Co-producing Psychiatric Education with Service User Educators: a Collective Autobiographical Case Study of the Meaning, Ethics, and Importance of Payment

**DOI:** 10.1007/s40596-021-01471-6

**Published:** 2021-06-01

**Authors:** Sophie Soklaridis, Alise de Bie, Rachel Beth Cooper, Kim McCullough, Brenda McGovern, Michaela Beder, Gail Bellissimo, Tucker Gordon, Suze Berkhout, Mark Fefergrad, Andrew Johnson, Csilla Kalocsai, Sean Kidd, Nancy McNaughton, Charlotte Ringsted, David Wiljer, Sacha Agrawal

**Affiliations:** 1 The Centre for Addiction and Mental Health, Toronto, ON Canada; 2 University of Toronto, Toronto, ON Canada; 3 McMaster University, Hamilton, ON Canada; 4 Wilfred Laurier University, Waterloo, ON Canada; 5 Unity Health Toronto, Toronto, ON Canada; 6 Sunnybrook Health Sciences Centre, Toronto, ON Canada; 7 University Health Network, Toronto, ON Canada; 8 Aarhus University, Aarhus, Denmark; 9 Toronto, ON Canada; 10 Mississauga, ON Canada

**Correction to: Academic Psychiatry (2020) 44:159–167**

10.1007/s40596-019-01160-5

This article was updated to correct co-author Gail Bellissimo’s last name.

